# Supplementary material for: Robust odor identification in novel olfactory environments in mice
Source: Nat Commun. 2023 Feb 13;14:673. doi: 10.1038/s41467-023-36346-x (PMC9925783; doi:10.1038/s41467-023-36346-x)
Supplement: Supplementary file 1 — Supplementary Information [file 41467_2023_36346_MOESM1_ESM.pdf]

Supplementary Note 1: Physical characteristics of drawn ROIs

The average area of the drawn ROIs was 1030.7±7.9 μm^2 (mean±s.e.m, n=5590 drawn ROIs, 11 WT mice). To determine whether the responses of single ROIs did not include mixed signals originating from multiple glomeruli, we analyzed the responses from nearby ROIs. Out of 35594 simultaneously recorded ROI pairs, there were 351 ROI pairs whose centers were closer than 50 µm or less. These neighbor ROIs could potentially include an overlap of signals originating from neighbor glomeruli. However, these neighbor ROIs responded to different sets of odors, with each pair of ROIs having 7.6± 3.1 odors (mean±s.d , 351 ROI pairs) with significant responses (z_score of -0.42 or less) for one ROI and not the other. There was only 1 pair of neighbor ROIs that were responsive to the same set of odors. Responses of neighbor ROIs were different, indicating that their signals originated from different glomeruli.

Supplementary Note 2: Changes in odor evoked z-score detection threshold did not affect algorithms performance

The performance of the SVM, logistic regression, and the NNC on novel background odors were resilient to changes in the threshold that determined whether an odor-ROI response was included We have used a z-score threshold of $\sigma_{\text{noise}}/\sqrt{n}$ =-0.42. The z-score threshold was based on the baseline imaging noise σ_{noise}, as well as the number of odor presentations that were used to calculate the average glomerular activation for an odor. We included 72885 odor-ROI responses (from a total of 120720 possible odor-ROI response to mixtures, 6 WT animals, 32 recording sessions). We have systematically changed the threshold of the ROI responses with 9 values between -0.1800 (most permissive) to -0.66(most restrictive) and repeated the analysis of figure 3. The most permissive threshold increased the number of odor-ROI responses by 17% whereas the most restrictive threshold decreased the number of odor-ROI responses by 16%. These changes in the number of odor-ROI responses resulted in no significant changes in the odor identification for the three considered algorithms in the presence of novel background odors compared to the performances using the z-score threshold of -0.42 ($p>0.4$, t-test, n=32 recording sessions).

Supplementary Note 3: Familiarity with background odor did not increase target recognition in novel backgrounds for WT mice

We wondered if WT mice could boost their performance on a novel background odor by having previous substantial exposure to that background odor outside the context of the task. During the task, animals got a total exposure to a novel background odor of at most 8 trialsx3.75 s=30 s. In order to increase the exposure time to a novel background odor, we divided our set of novel background odors into 2 odor groups, an exposed odor group (Group E) and a non-exposed odor (Group NE). Odor Group E consisted of 5 background odors (butyl propionate, ethyl tiglate, γ-terpinene, heptanal, and acetal) and the performance of our initial group of animals was 78.8% (4 animals, 132 trials). Odor Group NE consisted of six odors (2-3 pentanedione, ethyl benzoylacetate, hexanal, methyl pyruvate, 1-4 cineole, 4-methylanisole) that had a performance of 86.9% (4 animals, 176 trials). Before the start of behavioral training, a new group of 5 animals was pre-exposed to the odors in group E (5minutes x 5days=25 minutes total). There was only a 0.8% increase in the performance of the exposed odors (group E, 79.6%, 211 trials), which was not significant (p=0.89, Fisher exact test). The main effect was a reduction of the performance of the non-exposed group NE (75.2%, 250 trials).

Supplementary Note 4: Briefer inhalation widths were correlated with increased WT mice performance in novel environments

WT mice increased their sniff rate and reduced the inhalation width on the second sniff after the onset of the novel background odor, as they acquired the information that a novel odor was present from the first inhalation and reacted to it on the second inhalation. We compared the second inhalation width after the onset of the novel background odor between correct and incorrect trials to determine if fast sniffing was correlated with better performance. This second sniff width was significantly shorter (p=0.014, Wilcoxon rank sum test, single tailed) in the correct trials (91.3±2.0 ms, 552 trials, 8 WT mice, mean±s.e.m) compared to the incorrect trials (102.1±4.5 ms,131 trials). Interestingly, fast sniffing was not correlated with better performance for known backgrounds; the second inhalation width after (s)-(−)-limonene onset was not significantly shorter for known backgrounds (p=0.97, Wilcoxon rank sum test, single tailed), with a width of 101.6 ± 1.0 ms (n=2816 trials) for the correct trials and 94.6±2.7ms, (n=331 trials) for the incorrect trials.

We wondered if the correlation between the second sniff width and performance might be caused by slow fluctuations in animal alertness over the course of a behavioral session. If so, the trend between sniff rate and performance should already be noticeable from the first sniff after novel odor background onset. However, there was no difference in inhalation width (p=0.50, single trailed Wilcoxon sum test) between correct trials (110.2 ± 2.6 ms, 552 trials) and incorrect trials (109.6± 5.3 ms, 131trials). There was also no difference (p=0.85, Wilcoxon rank sum test, single tailed) for the known backgrounds, with 105.3±1.1ms (2816 trials) for the correct trials and 100.7±2.8 ms (331trials) for the incorrect trials. Faster sniffing increased odor identification performance only for novel background odors in WT mice.

Supplementary Note 5: Increased trial-to-trial variability did not affect NNC and linear classifier performance using real mixtures

We tested the effect of the increased variability in odor identification in novel environments using average glomerular responses from WT mice using real mixture combined with the increased CV_{uncorr} and the lower z-score threshold of -0.46 of *Cntnap2*$^{-/-}$ mice. The increased variability did not significantly affect the performance of SVM, logistic regression, and NNC algorithms on novel background odors as well as standard background odors. The performance of the logistic regressor for novel background odors in the increased variability regime (*Cntnap2*$^{-/-}$ like) was 68.2±2.1% (mean ± s.e.m, n=32 recording sessions) and it was not significantly different from the performance of the reduced variability mode (WT-like, 70.1±1.4%, mean ± s.e.m, p=0.56, t-test). The performance for novel background odors for the SVM linear classifier in the *Cntnap2*$^{-/-}$ like high-variability regime was 67.6±2.0% and it was not significantly different than the performance of the WT-like regime (69.8±2.3%, n=32 recording sessions, p=0.47, t-test). The performance of the NNC in the *Cntnap2*$^{-/-}$ like regime was 69.4±2.5% and it was not significantly different from the performance of the WT mice (70.8± 2.9%, p=0.71, t-test).

Supplementary Table 1: Animals used for behavior

Number of female mice	Genotype	Back.and target presentation (Async/sync)	Preexposure to background odors	Number of mixtures used in training set	Background used during training	Figure used
4	WT	Async.	No	16	(S)-(−)-Limonene	5,7,10
5	WT	Sync.	No	16	(S)-(−)-Limonene	6,10
5	WT	Async.	Yes	16	(S)-(−)-Limonene	-
3	WT	Async.	No	8	(S)-(−)-Limonene	7,10
4	*Cntnap2*$^{-/-}$	Async.	No	8	(S)-(−)-Limonene	10
3	WT	Async.	No	8	Butyl propionate	10
2	*Cntnap2*$^{-/-}$	Async.	No	8	Butyl propionate	10

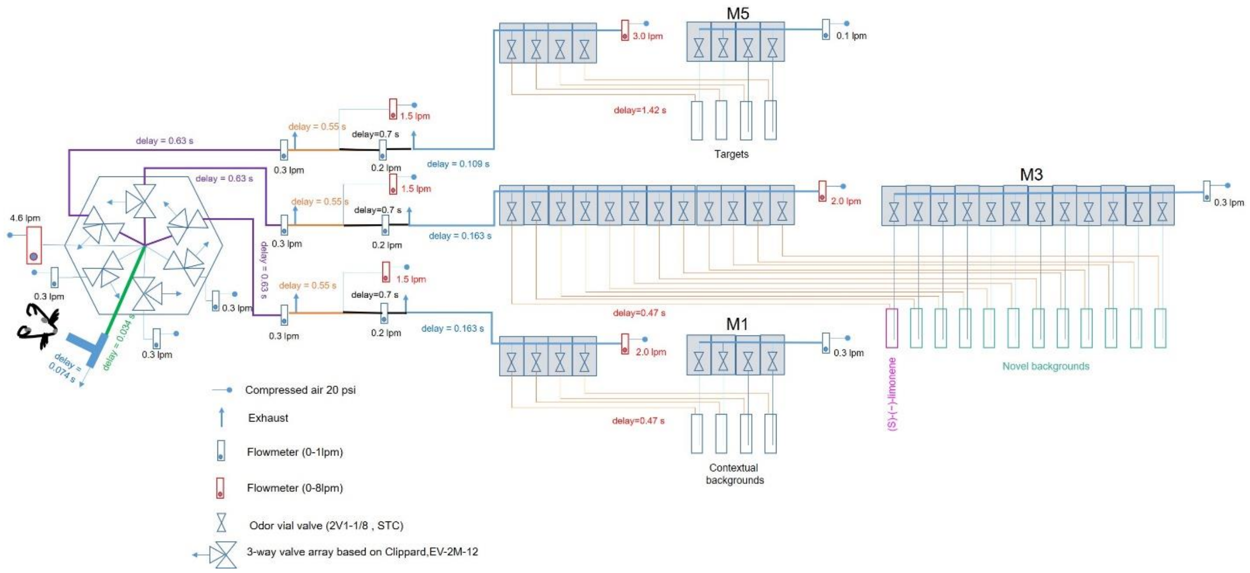

Supplementary Figure 1

Diagram of the triple serial air dilution odor machine. Values indicate the calculated delay on each line based on the tubing cross-section (1/8 inch diameter, Teflon coated) and airflow. The values of the flowmeters (and calculated delays) correspond to the concentrations used during testing with novel background odors. The total concentration of the targets at the animal snout was 0.025% of saturated vapor and the concentration of the contextual background odors and the novel odors, and (s)-(−)-limonene was 0.1% of saturated vapor.

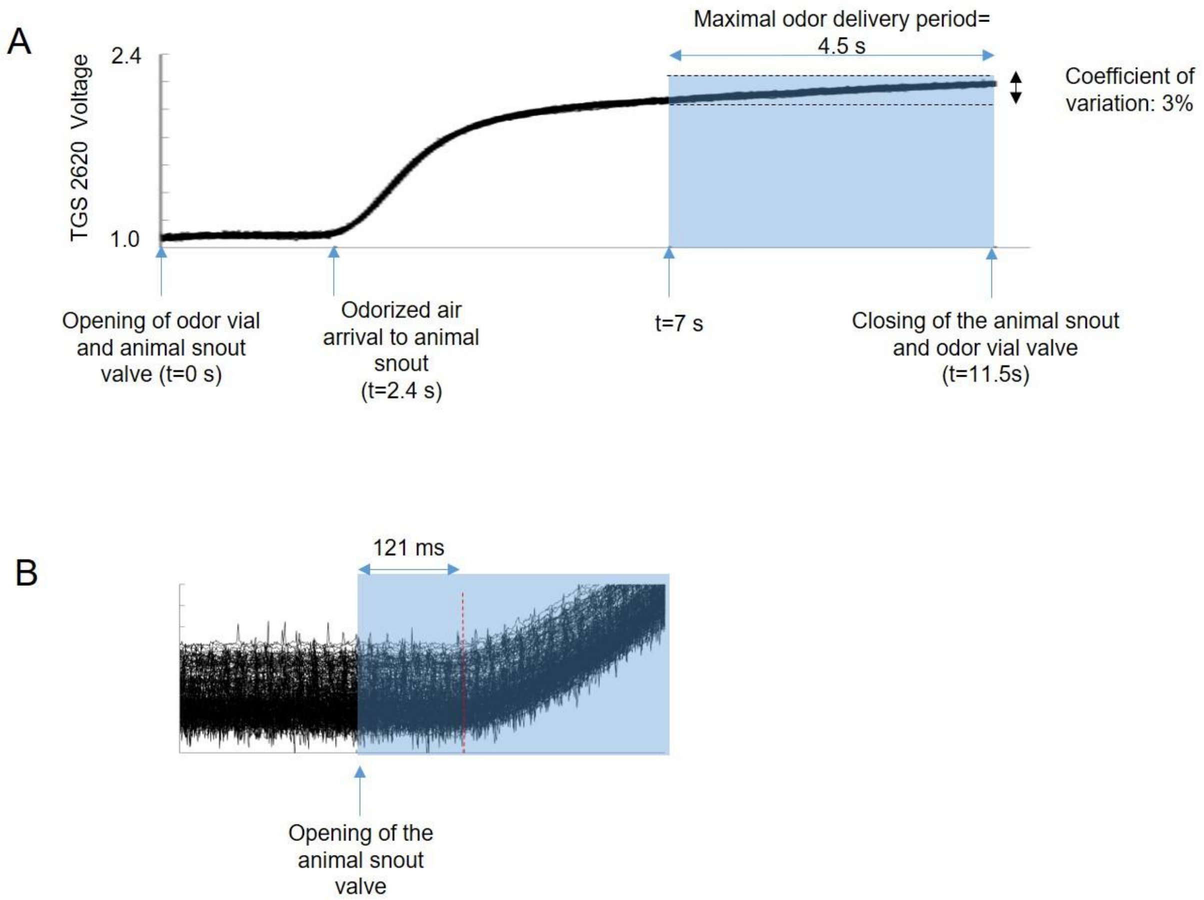

Supplementary Figure 2

Metal oxide sensor readings of the odor machine response time. **A.** Measurement of the delay between odor vial opening and odor arrival to the animal snout. The figure shows the superposition of 60 traces produced by 11.5 second long pulses of ethanol at 0.1% dilution connected to the contextual background line. To perform the measurement both the odor vial and the animal's snout valve opened synchronously. During the behavior and the imaging sessions, the odor vial valves opened at least 7 seconds before the animal snout valve to create a stable odor stream close to the animal snout. The calculated delay for this line to reach from the odor vial to the animal snout is 2.6 s. The actual measured delay was 2.4 s. We calculated a coefficient of variation of 3% over 60 repeats, by comparing the standard deviation with respect to the mean value of the signal averaged over the 4.5 seconds during the odor delivery. **B.** Measurement of the delay between animal snout valve opening and odor arrival to the animal snout. To perform this measurement, the odor vial valve opened 7 seconds before the animal snout valve opened to establish a constant odor concentration at the input of the animal snout valve. The calculated delay to reach from the animal snout valve to the animal snout is 108 ms. The actual measured delay was 121 ms.

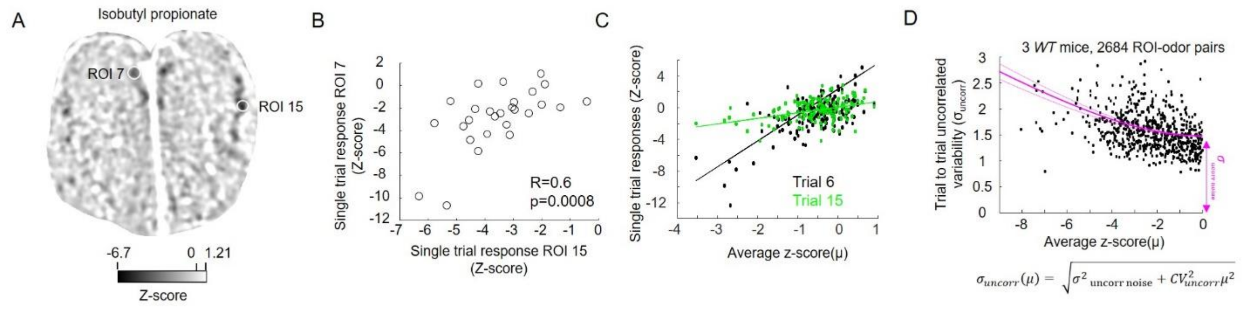

WT

Supplementary Figure 3

Intrinsic glomerular response in awake WT mice had correlated fluctuations. A. Example of an image of average z-score responses to 27 presentations of a mixture of ethyl caproate (0.1%), (s)-(−)-limonene (0.1%), and isobutyl propionate (0.025%) in an awake WT mouse. The odor was presented as a 9 second pulse. Odor responses are the average z-score calculated using the period between 2 and 9 seconds following odor onset and using the 5 seconds preceding the onset of the odor as the baseline. **B.** Example of trial by trial correlated odor evoked responses of two glomeruli. Linear correlation was significant (R=0.66, p=0.0008, Pearson linear correlation coefficient). **C.** Glomerular responses for 156 glomeruli for 2 different odor mixture presentations plotted against the average response over 27 odor presentations. Solid lines are the least square linear fits. Glomerular odor response variations had population correlated fluctuation and an uncorrelated fluctuation. The population correlated fluctuation is given by the least squares linear fits, whereas deviation from the least squares linear fits correspond to the uncorrelated fluctuation. **D.** We calculated the deviations from the least squares fitted line for 2684 ROI-odor pairs recorded from 3 WT mice and plotted the standard deviations of their deviation from the best fitted line (σ_{uncorr}) against the average response for that ROI-odor pair (μ). The total variance σ^2_{uncorr} is given by the sum of an odor independent variance $\sigma^2_{\text{uncorr noise}}$ and a response dependent variance. The purple line is the fitted line and the dotted lines are the 95% confidence interval of the fit.

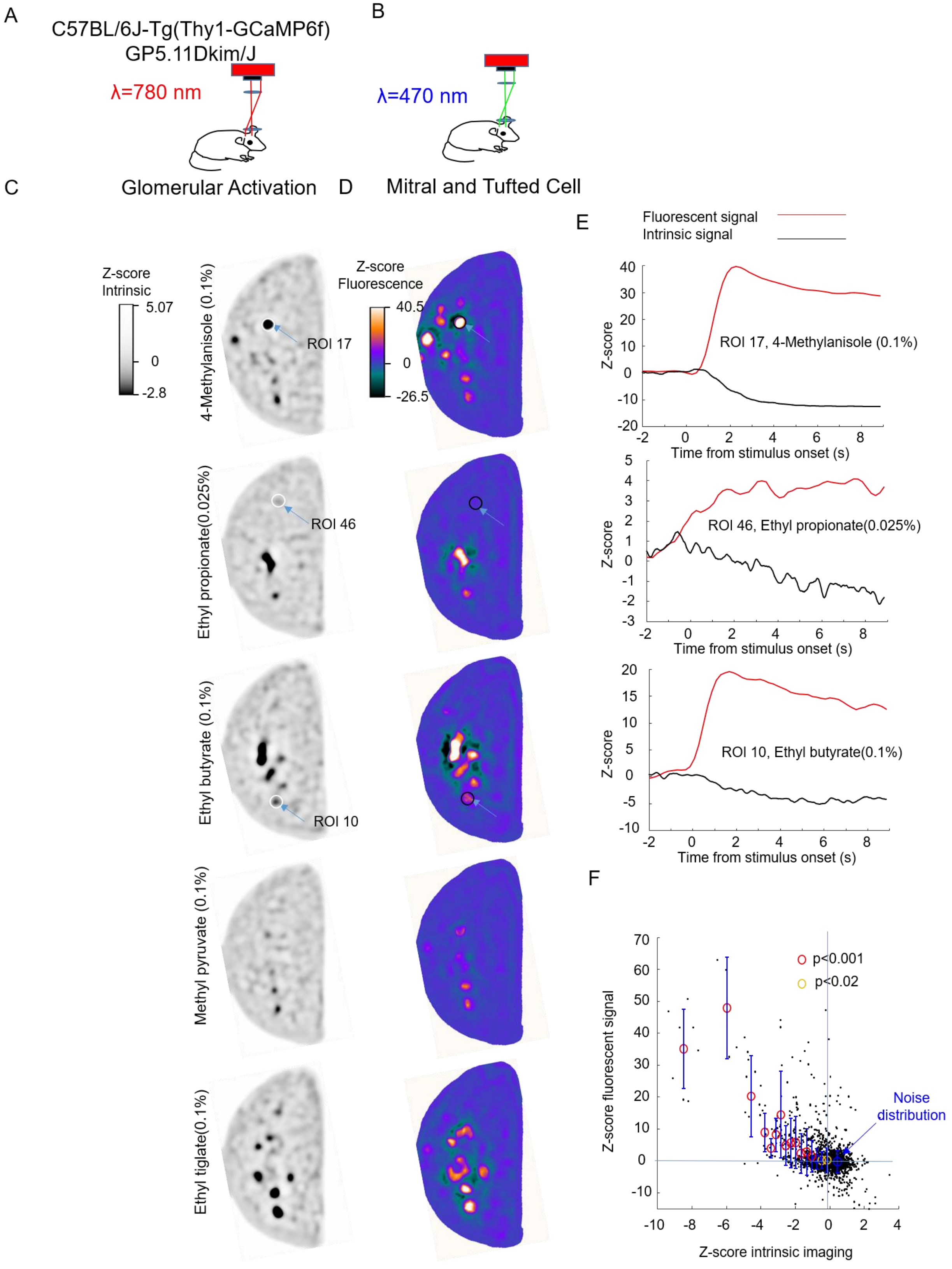
A
C57BL/6J-Tg(Thy1-GCaMP6f)
GP5.11Dkim/J
λ=780 nm
B
λ=470 nm
C
Glomerular Activation
D
Mitral and Tufted Cell
E
Fluorescent signal
Intrinsic signal
Z-score
Intrinsic
5.07
0
-2.8
Z-score
Fluorescence
40.5
0
-26.5
4-Methylanisole (0.1%)
ROI 17
Ethyl propionate(0.025%)
ROI 46
Ethyl butyrate (0.1%)
ROI 10
Methyl pyruvate (0.1%)
Ethyl tiglate(0.1%)
ROI 17, 4-Methylanisole (0.1%)
ROI 46, Ethyl propionate(0.025%)
ROI 10, Ethyl butyrate(0.1%)
Z-score
Time from stimulus onset (s)
F
p<0.001
p<0.02
Noise
distribution
Z-score fluorescent signal
Z-score intrinsic imaging

Supplementary Figure 4

Glomerular activation measured with intrinsic signal correlate with increases in fluorescence in Thy1-GCaMP6f mouse. We implanted a C57BL/6J-Tg(Thy1-GCaMP6f) GP5.11Dkim/J(Dana et al. 2014) mouse with a window over the olfactory bulb as described above. **A.** We presented 20 odors using 9 second odor pulses at the concentrations used for the behavior in an awake mouse and recorded intrinsic signal using 780 nm infrared light. **B.** We also used a 470 nm blue light and recorded the green fluorescent responses that correspond to mitral and tufted cells, the output neurons of the olfactory bulb. **C,D.** Examples of average z-score of the intrinsic imaging averaged over 2 to 9 seconds from odor onset. The negative deflections in odor evoked glomerular responses in the intrinsic signal colocalized with odor evoked positive deflections in the fluorescence signal. The fluorescent signal had larger magnitude compared to the intrinsic signal. **E**. Examples of time course responses from individual glomeruli to 9 second odor pulses that start at 0 s. Even intrinsic signals with relatively small z-scores (see ROI 46 in response to ethyl propionate) resulted in increases in the fluorescence signal. **F.** Relationship between the ROI-odor intrinsic signal, defined in the intrinsic signal, and the fluorescence signal. Black dots represent individual ROI-odor pairs and circles coordinates are the mean of fluorescence and intrinsic signal, binned according to the z-score of the intrinsic signal. The error bars are the standard deviation of the fluorescence signal of the ROI-odor pairs in a bin. We compared the fluorescence signal of each bin with the distribution of fluorescence in the absence of odor responses (noise distribution) using a two-tailed t-test. The responses were significantly different from the noise distribution for responses with a mean z-score response of intrinsic imaging of -0.3 ($p<0.02$) or larger.

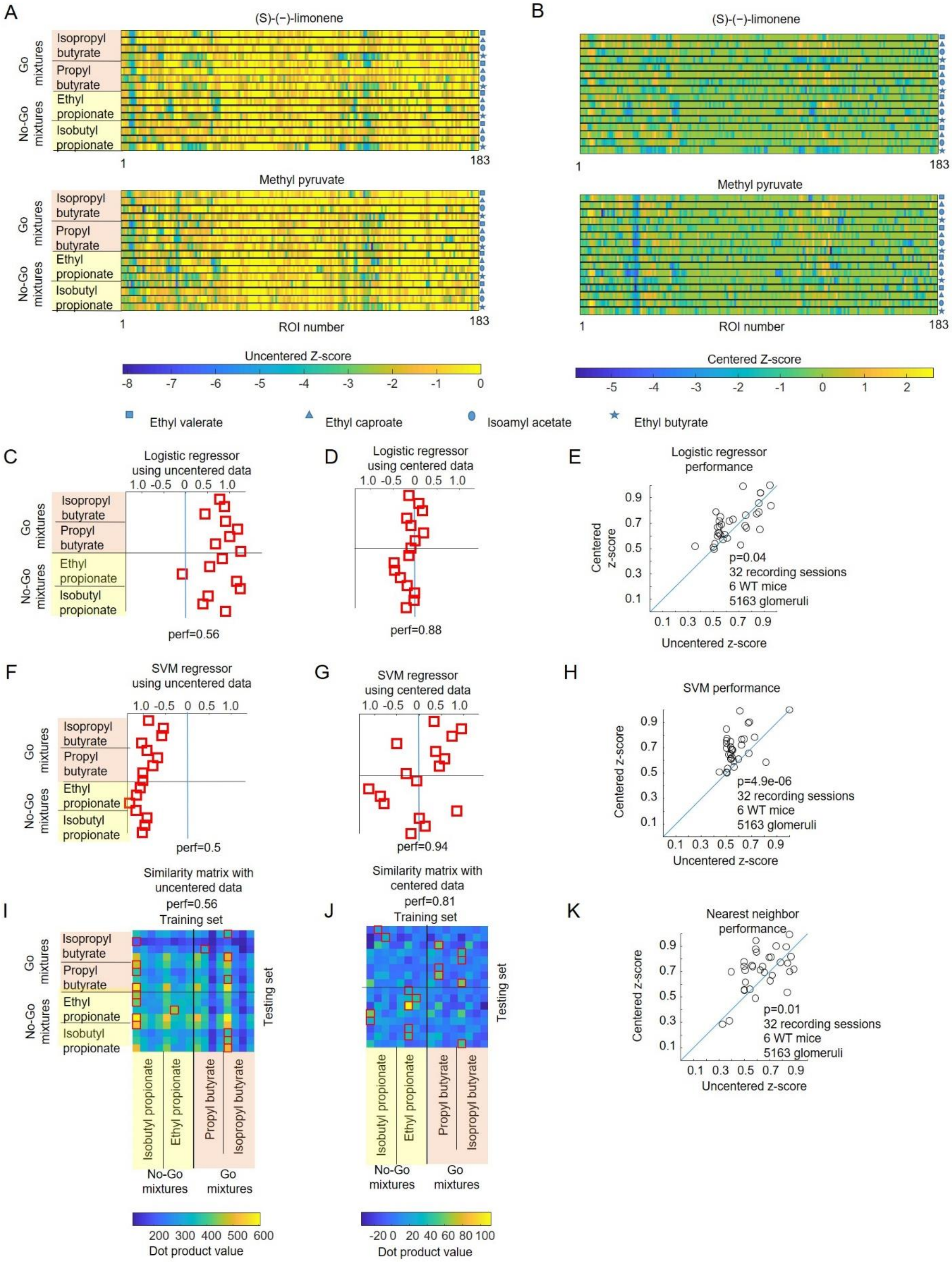

A
(S)-(−)-limonene
Isopropyl butyrate
Propyl butyrate
Ethyl propionate
Isobutyl propionate
Go mixtures
No-Go mixtures
1
183
Methyl pyruvate
ROI number
Uncentered Z-score
-8 -7 -6 -5 -4 -3 -2 -1 0
B
(S)-(−)-limonene
Methyl pyruvate
ROI number
Centered Z-score
-5 -4 -3 -2 -1 0 1 2
Ethyl valerate
Ethyl caproate
Isoamyl acetate
Ethyl butyrate
C
Logistic regressor using uncentered data
perf=0.56
D
Logistic regressor using centered data
perf=0.88
E
Logistic regressor performance
Centered z-score
Uncentered z-score
p=0.04
32 recording sessions
6 WT mice
5163 glomeruli
F
SVM regressor using uncentered data
perf=0.5
G
SVM regressor using centered data
perf=0.94
H
SVM performance
p=4.9e-06
32 recording sessions
6 WT mice
5163 glomeruli
I
Similarity matrix with uncentered data
perf=0.56
Training set
Testing set
Isobutyl propionate
Ethyl propionate
Propyl butyrate
Isopropyl butyrate
No-Go mixtures
Go mixtures
200 300 400 500 600
Dot product value
J
Similarity matrix with centered data
perf=0.81
Training set
Testing set
-20 0 20 40 60 80 100
Dot product value
K
Nearest neighbor performance
p=0.01
32 recording sessions
6 WT mice
5163 glomeruli
Centered z-score
Uncentered z-score

Supplementary Figure 5

Centering of the glomerular data improved the performance of linear classifiers and NNC for odor identification in novel background odors. **A.** Example of uncentered z-score responses of the training set and the test set in a recording session of a WT mice where the novel background odor was methyl pyruvate. **B.** Centered z-score, where for each glomerular response we subtracted the average response of that glomerulus to the training set. **C.** Performance of the logistic regressor linear classifier trained and tested with the uncentered z-score. The red squares are the output of the linear classifier with the mixtures with novel background odors. Performance was close to chance. **D.** Performance was higher when using the centered z-score for training and testing. **E.** Performance of logistic regressor calculated using the uncentered data versus the centered data on novel background odors. Each symbol corresponds to the performance on one recording session (n=32 recording sessions from 6 animals, 5163 glomeruli). The performance of the logistic classifier was significantly higher (paired two-tailed t-test) when using the centered data. **F-G-H.** SVM linear classifier also had better performance when using centered z-score data. P-value was calculated using a paired t-test. **I-J** Effect of centering on the NNC. Example of matrix of similarities (dot products) between the training set and the test set calculated using the uncentered (**I**) and centered (**J**) z-score. For each mixture in the training set, the red square indicates the location of the most similar mixture from the training set. The dot product matrix using the uncentered data produced larger and less differentiated values. The most similar mixtures for the uncentered data were not located on the correct discrimination quadrants (right-up for the go mixtures and left-down for the no-go mixtures). **K.** The performance of the NNC was significantly higher (paired two-tailed t-test) when using the centered data.

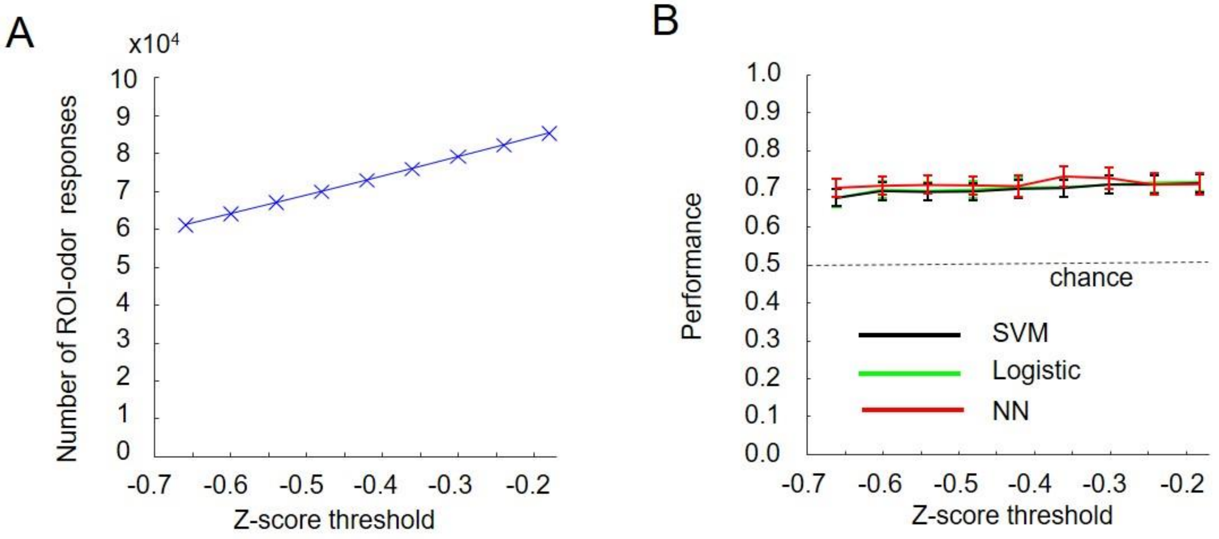

Supplementary Figure 6

Performance of linear classifiers and NNC for odor identification in novel background odors were robust to changes in the threshold of glomerular responses using intrinsic imaging. **A.** Average number of glomeruli-odor responses included per recording session as a function of the threshold of z-score responses. Error bars are the s.e.m for 32 recording sessions (6 WT mice, 5163 ROI). The number of ROI-odor responses fluctuated between -16% and +17% as the threshold for z-score responses was changed between -0.18 (most permissive) to -0.66(most restrictive). The reference z-score threshold was -0.42 which resulted in 72885 odor-ROI responses. **B.** Performance on individual recording sessions of the linear classifiers and NNC as a function of the z-score threshold. Error bars are s.e.m for 32 recording sessions. The performance of the NNC, and the linear SVM and logistic regression were not affected by changes in the threshold of detection of the z-score.

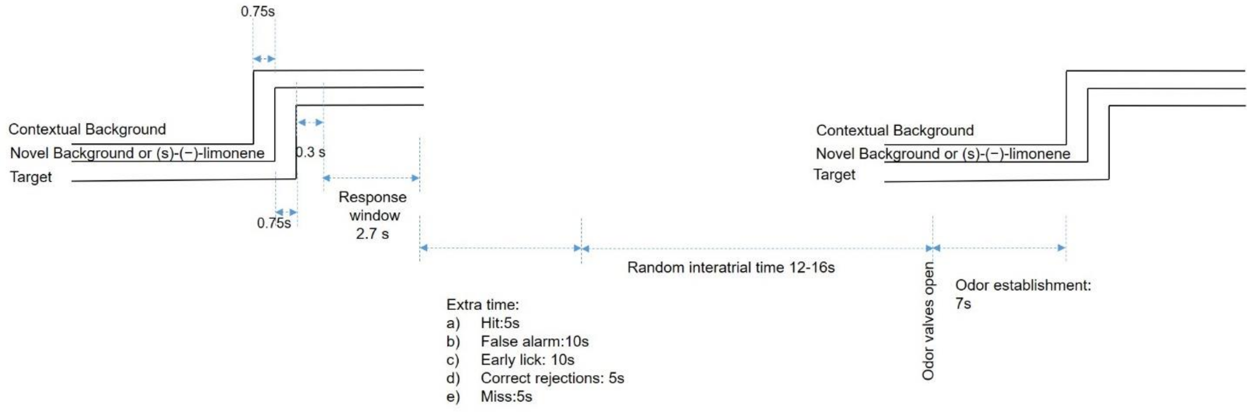

Supplementary Figure 7

Temporal profile of olfactory stimulus delivery and behavioral contingencies for the asynchronous task. Odor valves opened for 7 seconds to establish stable odor concentration at the input of the valves close to the animal's snout. After 7 seconds, a valve near the mouse opened and routed the contextual background odor flow to the mouse's snout. After 0.75 s, the novel background odor was delivered, followed after 0.75 s by the target odor. The mouse had to lick with a latency of >0.3 s from target onset to get rewarded. The target appeared concurrently with the background odors for 3 s. If the target was a go-odor and the animal licked during the response window (hit), the animal received water and 5 seconds were added to the intertrial interval. If the target odor was a no-go odor and the animal responded, it was a false alarm and 10 s were added to the intertrial interval. If the animal responded before the 0.3 s minimal latency from target onset (early lick), extra 10 s were added to the intertrial interval. If the animal did not lick in response to the odor, either as a miss or a correct rejection, 5 s were added to the intertrial interval. There was an extra random interval between 12 and 16 s that was always present in the intertrial interval.

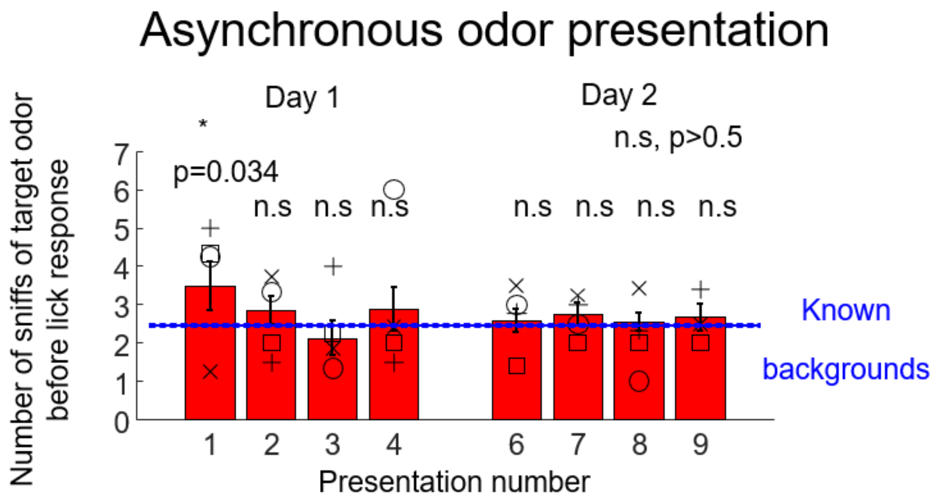

Supplementary Figure 8

Number of sniffs before a lick response in response to the target odor were elevated in the first presentations of the novel background odors for WT mice with asynchronous odor presentation, where the target odor onset was preceded by 1.5 s of contextual background odor and 0.75 s of novel background odor. Number of sniffs taken as a function of the times that a novel background odor has been presented. WT mice increased the number of sniffs on the first presentation and on subsequent presentations values dropped to values closer to 3 sniffs, the number of sniffs used in known backgrounds (all values are mean±s.e.m, with n=13 lick responses). The horizontal solid and dotted blue lines represents the number of sniffs (mean±s.e.m) taken for lick responses to targets in the presence of known background odors. The statistical tests compared the number of sniffs for novel background odors for a given presentation number with the number of sniffs taken for known background odor using a Bonferroni-corrected two-tailed t-test. Individual symbols represent average values for individual animals.

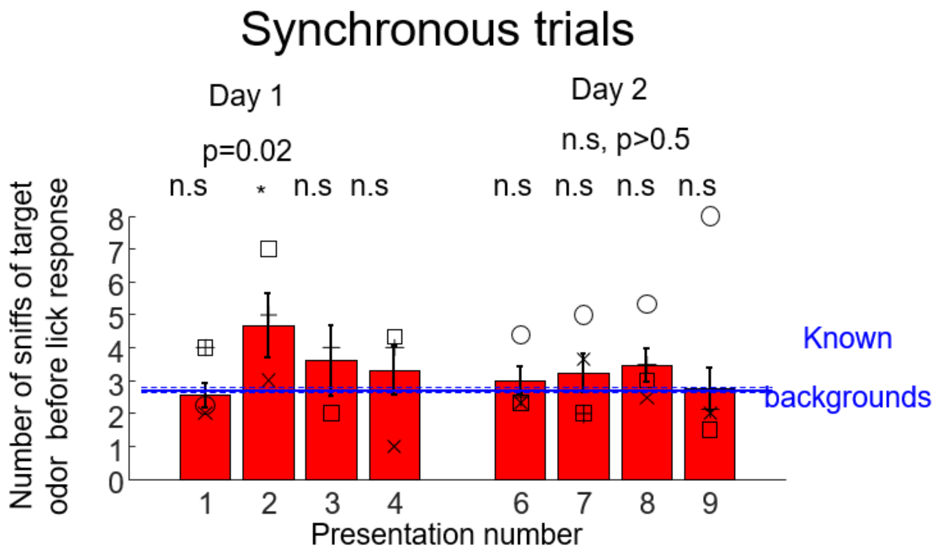

Supplementary Figure 9

Number of sniffs before a lick response in response to the target odor were elevated on the second presentation of the novel background odors for WT mice with synchronous odor presentation, where the target odor onset was preceded by only 50 ms of contextual background odor and novel background odor. Number of sniffs taken as a function of the times that a novel background odor has been presented. WT mice increased the number of sniffs on the second presentation and on subsequent presentations values dropped to values closer to 3 sniffs, the number of sniffs used in known backgrounds (all values are mean±s.e.m). The horizontal solid and dotted blue lines represents the number of sniffs (mean±s.e.m, n= 519 licking trials) taken for lick responses to targets in the presence of known background odors. The statistical tests compared the number of sniffs for novel background odors for a given presentation number with the number of sniffs taken for known background odor using a Bonferroni-corrected two-tailed t-test. Individual symbols represent average values for individual animals.

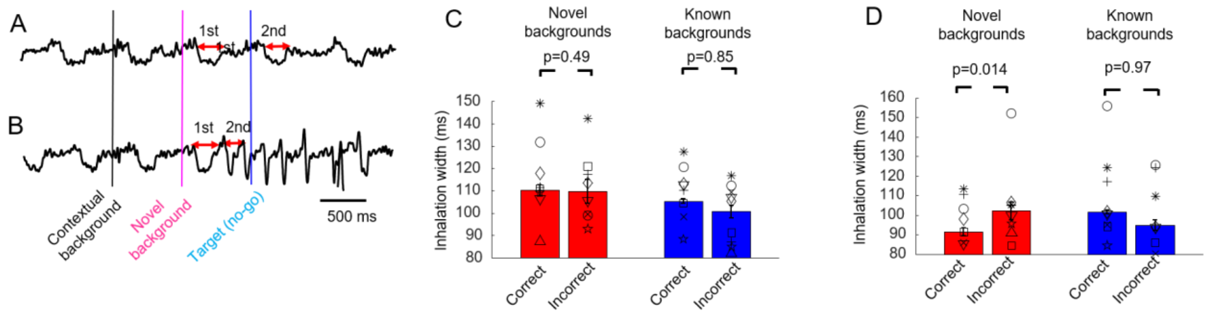

Supplementary Figure 10

Brief inhalations in response to novel background odors were associated with improved performance **A.** Example trial of sniffing response to a novel background in which the WT mouse did not change their inhalation widths nor frequency (downward deflections) in response to the novel background odor. **B.** Example trial of sniffing response where the mouse produced a brief second inhalation after the onset of the novel background. **C.** Mean±s.e.m of inhalation widths. The first inhalations in response to novel backgrounds and known backgrounds were not significantly different between correct and incorrect trials. P-values for the reduction of the sniff widths were calculated using the single tailed Wilcoxon rank sum test. **D.** The second inhalation width reflects the animal reaction to novel background odors and it was significantly briefer in correct trials. There was not difference in inhalation width between correct and incorrect for known backgrounds. P-values for the reduction of the sniff widths were calculated using the single tailed Wilcoxon rank sum test. See **Supplementary Note 4** for details.

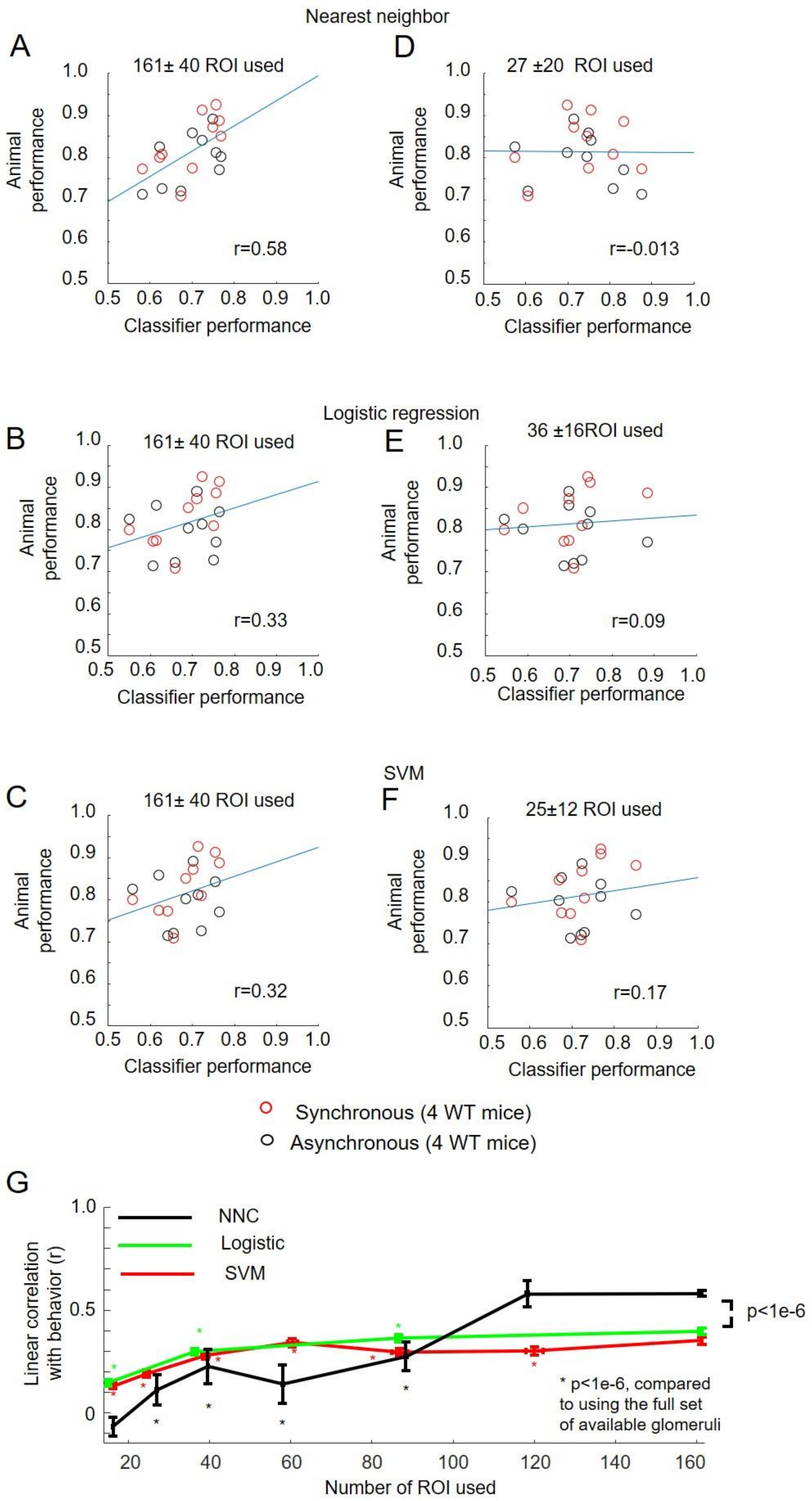

Nearest neighbor
A
161± 40 ROI used
r=0.58
D
27 ±20 ROI used
r=-0.013
Logistic regression
B
161± 40 ROI used
r=0.33
E
36 ±16ROI used
r=0.09
SVM
C
161± 40 ROI used
r=0.32
F
25±12 ROI used
r=0.17
Animal performance
Classifier performance
Synchronous (4 WT mice)
Asynchronous (4 WT mice)
G
NNC
Logistic
SVM
p<1e-6
* p<1e-6, compared to using the full set of available glomeruli
Linear correlation with behavior (r)
Number of ROI used

Supplementary Figure 11

Classifiers that included more glomeruli had higher correlation with animal behavior. **A.** Performance of the NNC for 10 novel background odors was calculated using all the available glomeruli per imaging session (161±40 ROI, mean±s.d, 32 imaging sessions, 6 WT mice). Black circles *y* coordinates represent the average behavioral performance on a novel background odor from four WT mice that performed the asynchronous task and red circle1 *y* coordinate represent the average performance of the four WT mice that performed the synchronous task. The blue line is the linear correlation between imaging data and behavior. **B-C** Similar plot for logistic regression and SVM. **D-F. Reducing the number of glomeruli reduced correlation with behavior for all three classifiers. G.** Classifier's correlation with behavior as a function of the number of ROI included. Error bars represent the standard deviation of the distribution of linear correlation coefficients (vertical) and number of glomeruli(horizontal) included calculated using a Montecarlo simulation with 500 repeats. The asterisks indicate the results of two-tailed t-test comparing the distribution of correlations using all available glomeruli against the distribution of correlations using the reduced number of glomeruli.

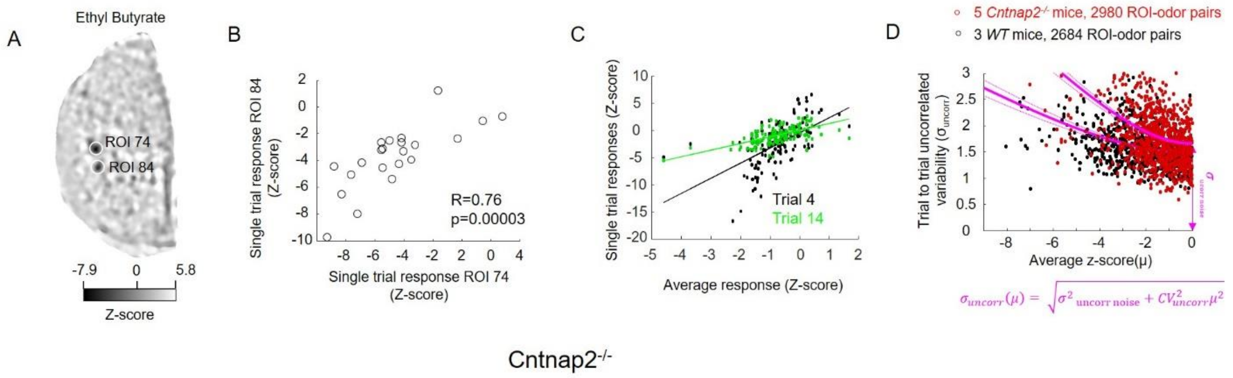

$$\sigma_{uncorr}(\mu) = \sqrt{\sigma^2_{\text{uncorr noise}} + CV^2_{uncorr}\mu^2}$$

Supplementary Figure 12

Intrinsic glomerular responses in awake *Cntnap2*$^{-/-}$ mice had correlated fluctuations. **A.** Example of an image of average z-score responses to 22 presentations of ethyl butyrate (0.1%) in an awake *Cntnap2*$^{-/-}$ mouse. The odor was presented as a 9 second pulse. Odor responses are the average z-score calculated using the period between 2 and 9 seconds following odor onset and using the 5 seconds preceding the onset of the odor as the baseline. **B.** Example of trial by trial correlated odor evoked responses of two glomeruli located on different spots. Linear correlation was significant (R=0.76, p=0.00003, Pearson linear correlation coefficient). **C.** Glomerular responses for 132 glomeruli for 2 different odor mixture presentations plotted against the average response over 22 odor presentations. Solid lines are the least square linear fit. **D.** We calculated the deviations from the best fitted line for 2980 ROI-odor pairs recorded from 5 *Cntnap2*$^{-/-}$ mice and plotted in red the standard deviations of their deviation from the best fitted line (σ_{uncorr}) against the average response for that ROI-odor pair (μ). The black dots correspond to data from the WT mice. Purple line indicates mean fitted trial-to-trial uncorrelated variability and dotted lines are the 95% confidence intervals for both genotypes. *Cntnap2*$^{-/-}$ mice had higher uncorrelated trial-to-trial variability compared to WT mice.

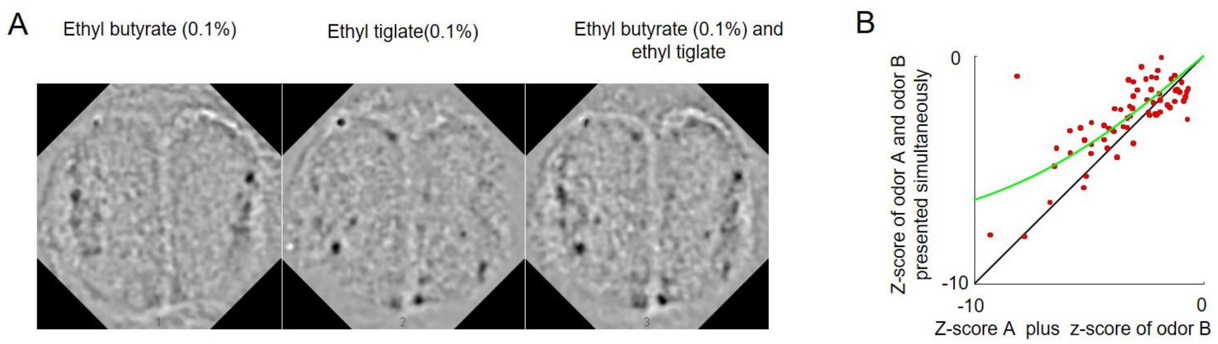

Supplementary Figure 13

Deviations of linear addition on imaging of odor mixtures **A.** Glomerular activation patterns of odors presented separately and combined together as a mixture approximated as a linear combination **B.** The main observed effect was sublinear and we approximated as a saturating nonlinearity.

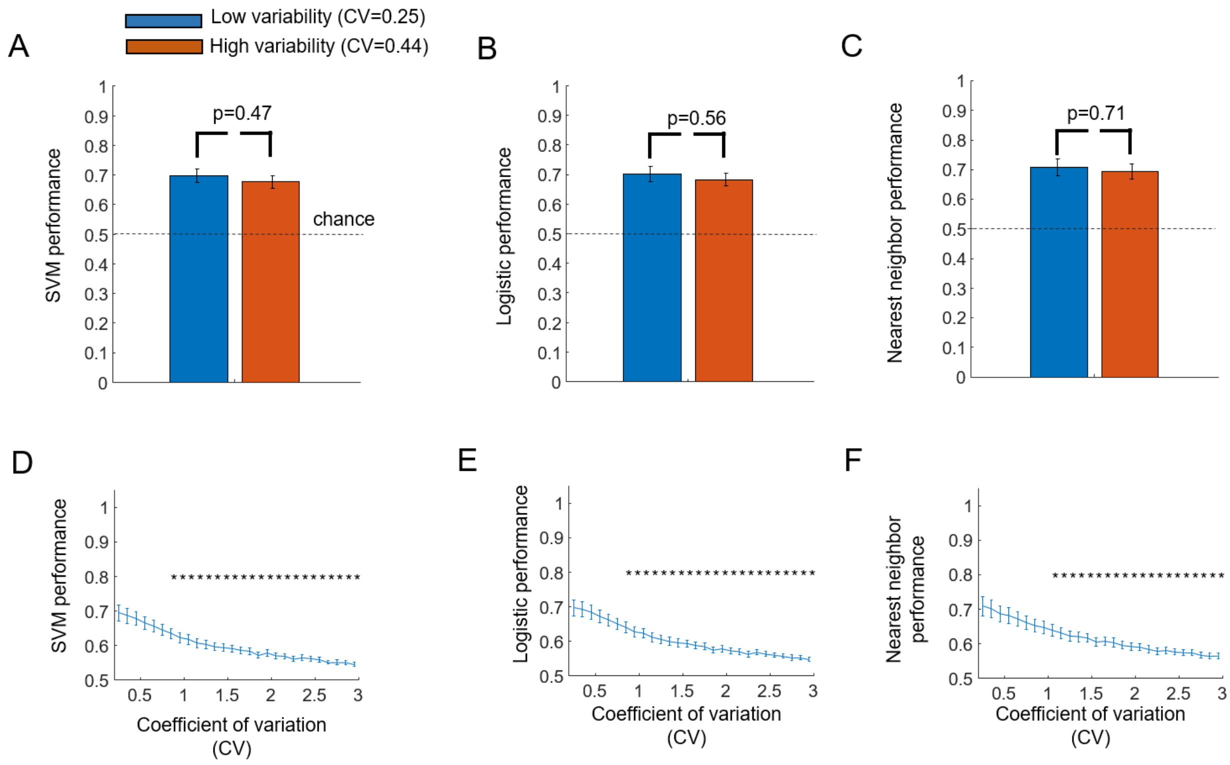

Supplementary Figure 14

Performances of the linear SVM, logistic, and NNC on novel background odors were not affected by the higher trial to trial variability seen in *Cntnap2*$^{-/-}$ mice. A. Average performance ± s.e.m. of linear SVM classifier in the low variability (CV=0.25, WT like) and high variability regime (CV=0.44, *Cntnap2*$^{-/-}$ like). The performance for each recording session was calculated by creating 100 instantiations of the test set (16 mixtures that included a novel background odor) as shown in Figure 3. To simulate the *Cntnap2*$^{-/-}$ mice data, we used the higher trial to trial variability (CV_{uncorr} = 0.44) and the lower z-score threshold (z-score<-0.46) whereas to simulate the WT mice we used the lower variability (CV_{uncorr} = 0.25) and the higher z-score threshold (z_score<-0.42). The p-values was calculated using a two-tailed t-test comparing the high variability and the low variability results (n=32 recording sessions, 6 WT mice data). **B.** Similar plot for the logistic model (n=32 recording sessions, 6 WT mice data). The p-value was calculated using a two-tailed t-test. **C.** The higher trial-to-trial variability also did not affect performance of the NNC (n=32 recording sessions, 6 WT mice data). The p-values was calculated using a two-tailed t-test. **D.** Average performance± s.e.m. of the linear SVM as a function of the coefficient of variation, which ranged between 0.25 (value measured in WT mice) to 2.95. The asterisks mark significant differences (p<0.05, two-tailed t-test, n=32 recording sessions) between the performance of the WT mice data (CV=0.25) with the simulations with higher coefficients of variation. SVM performance decayed significantly when the CV was 0.85 or higher, which is almost two-fold the CV measured in *Cntnap2*$^{-/-}$ mice (CV=0.44). **E, F.** Similar plots for the logistic regression and the NNC (n=32 recording sessions, 6 WT mice data). The asterisks mark significant differences (p<0.05, two-tailed t-test, n=32 recording sessions) between the performance of the WT mice data (CV=0.25) with the simulations with higher coefficients of variation. Logistic regression

required a CV of 0.85 or higher for significant reduction in performance and NNC required a CV of 1.05 or higher to affect performance.

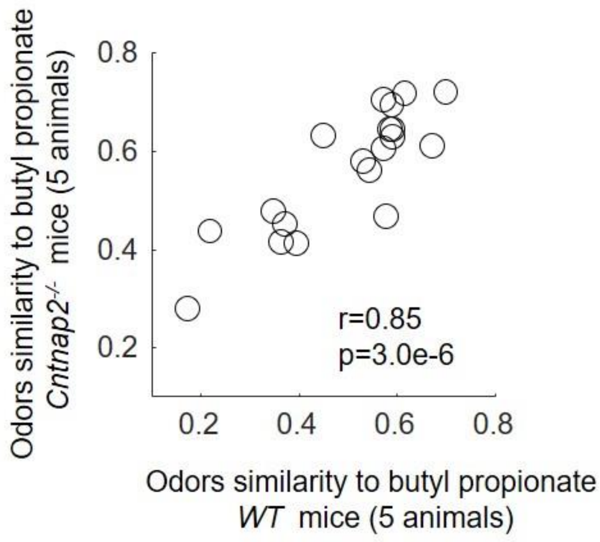

Supplementary Figure 15

Butyl propionate odor responses were similar for WT mice and *Cntnap2*$^{-/-}$ mice. The average glomerular response of butyl propionate was not significantly different (p=0.36, two-tailed t-test) in the *Cntnap2*$^{-/-}$ mice (-0.66±0.03 z-score, mean±s.e.m, 643 glomeruli, 5 *Cntnap2*$^{-/-}$ mice) and WT mice (-0.70±0.04 z-score, mean±s.e.m, 775 glomeruli, 5 *WT* mice). The fraction of glomeruli activated by butyl propionate was also not different between *Cntnap2*$^{-/-}$ mice and WT mice (*Cntnap2*$^{-/-}$ mice, 0.52±0.04, mean±s.e.m, 5 animals; WT mice, 0.53± 0.05, mean±s.e.m, p=0.86, two-tailed t-test). The figure shows that the similarity of butyl propionate with the other 19 odors used was highly correlated between *Cntnap2*$^{-/-}$ mice and WT mice.
