# Supplementary material for: Robust odor identification in novel olfactory environments in mice
Source: Nat Commun. 2023 Feb 13;14:673. doi: 10.1038/s41467-023-36346-x (PMC9925783; doi:10.1038/s41467-023-36346-x)
Supplement: Supplementary file 3 — Description of Additional Supplementary Files [file 41467_2023_36346_MOESM3_ESM.pdf]

Description of Additional Supplementary Files

Supplementary Data 1: Detailed training schedule
